# The Mechanosensing and Global DNA Methylation of Human Osteoblasts on MEW Fibers

**DOI:** 10.3390/nano11112943

**Published:** 2021-11-03

**Authors:** Pingping Han, Cedryck Vaquette, Abdalla Abdal-hay, Sašo Ivanovski

**Affiliations:** 1 Center for Oral-Facial Regeneration, Rehabilitation and Reconstruction (COR3), Epigenetics Nanodiagnostic and Therapeutic Group, School of Dentistry, The University of Queensland, Brisbane, QLD 4006, Australia; p.han@uq.edu.au; 2 School of Dentistry, The University of Queensland, Herston, QLD 4006, Australia; c.vaquette@uq.edu.au (C.V.); abdalla.ali@uq.edu.au (A.A.-h.); 3 Department of Mechanical Engineering, Faculty of Engineering, South Valley University, Qena 83523, Egypt

**Keywords:** microgeometry, mechanobiology, global DNA methylation, osteoblast mechanosensing

## Abstract

Cells interact with 3D fibrous platform topography via a nano-scaled focal adhesion complex, and more research is required on how osteoblasts sense and respond to random and aligned fibers through nano-sized focal adhesions and their downstream events. The present study assessed human primary osteoblast cells’ sensing and response to random and aligned medical-grade polycaprolactone (PCL) fibrous 3D scaffolds fabricated via the melt electrowriting (MEW) technique. Cells cultured on a tissue culture plate (TCP) were used as 2D controls. Compared to 2D TCP, 3D MEW fibrous substrates led to immature vinculin focal adhesion formation and significantly reduced nuclear localization of the mechanosensor-yes-associated protein (YAP). Notably, aligned MEW fibers induced elongated cell and nucleus shape and highly activated global DNA methylation of 5-methylcytosine, 5-hydroxymethylcytosine, and N-6 methylated deoxyadenosine compared to the random fibers. Furthermore, although osteogenic markers (*osterix-OSX* and *bone sialoprotein-BSP*) were significantly enhanced in PCL-R and PCL-A groups at seven days post-osteogenic differentiation, calcium deposits on all seeded samples did not show a difference after normalizing for DNA content after three weeks of osteogenic induction. Overall, our study linked 3D extracellular fiber alignment to nano-focal adhesion complex, nuclear mechanosensing, DNA epigenetics at an early point (24 h), and longer-term changes in osteoblast osteogenic differentiation.

## 1. Introduction

In the native bone milieu, osteoblasts can sense their local structural microenvironment to form focal adhesions and initiate “outside-in” mechanotransductive signals, leading to extracellular matrix formation along the cellular direction [1,2,3]. Biomaterial substrates with two dimensional (2D) or three dimensional (3D) structures as biophysical cues have a critical influence on mammalian cell phenotype, proliferation, adhesion, and differentiation [1,4,5,6,7]. Comprehensive studies have demonstrated that 2D substrates generate “outside-in” mechanotransductive signals that permit cells to sense and respond dynamically to their surrounding microenvironment [8,9]. Specifically, a 2D nanoscale grooved model established on titanium-alloy substrates by laser ablation demonstrated that the unique bone matrix formation orthogonal to mouse osteoblast cell alignment was facilitated by 3D nano-sized focal adhesion (FAs) maturation [2,3]. Furthermore, a previous study showed that 2D collagen hydrogels regulate histone modification and non-coding RNA [1]. Forces that are generated by nano-scaled FAs (such as vinculin [10]) are then transmitted to the cytoplasm, nucleus (via nuclear mechanosensor–YAP/TAZ [11]) and chromatin, through physical links on the nuclear membrane and the mesh-structured nuclear Lamin A/C network [12]. This process, in turn, may govern epigenetic mechanisms, as well as transcription activation and repression, thus influencing cell behavior and cell differentiation fate [1,13,14]. 

Epigenetics (including DNA methylation, histone modification, and non-coding RNA) refers to heritable changes in gene expression that do not involve changes to the DNA sequence, but rather act through chemical alterations of DNA that induce rapid inactivation or activation of genes [15,16]. DNA methylation involves the addition of methyl groups to either cytosine or adenine at specific sites in the DNA sequence and is dynamically regulated by methyl writing enzymes (DNA methyltransferases (DNMTs)) and methyl erasing enzymes (ten-eleven translocation methylcytosine dioxygenase (TETs)) [16,17]. The most common form of DNA methylation is 5 methylcytosine (5mC) and 5mC can be demethylated by TETs to become 5-hydroxymethylcytosine (5hmC). Beyond 5mC and 5hmC, N6-methyladenosine modification in DNA (m6dA) is the most common DNA modification. The evidence shows that DNA methylation is associated with osteogenic differentiation of mesenchymal stem cells [18,19] and periodontitis disease pathogenesis. [20] The concept of 2D biomaterial physical features acting as epigenetic cues has been proposed [13,21], thus, it is important to investigate the effect of a micro-scaled fiber arrangement (random or aligned) within the 3D structure of the biomaterials that can lead to DNA hypo-or hyper-methylation when compared to a 2D substrate.

The biomaterial-based 3D structure provides a closer representation of in vivo conditions compared with a conventional 2D system. A 3D poly(l-lactide-co-caprolactone) (PLCL) membrane with aligned nanofibers induced an increased histone H3 acetylation and H3 methylation compared to random fibers in mouse ear fibroblasts [22]. However, the sensing and response of human osteoblasts to micro-scaled fiber deposition (aligned and random) remain poorly understood. The present study aimed to investigate 3D mechanobiology in terms of focal adhesion and nuclear mechanotransduction, as well as global DNA epigenetics in human osteoblasts. In this study, a medical-grade polycaprolactone (PCL) fibrous scaffold with random and aligned fibers as 3D substrates was fabricated by melt electrowriting (MEW) technology. MEW precisely places continuous PCL fibers onto a collector plate, as dictated by computer-aided design software [23,24]. PCL was selected due to its relative abundance, low cost, low immunogenicity reactions [25,26,27], FDA approval for use in humans [24], and more recently, for its use in tissue regeneration applications. Our study not only provides new insights in understanding the roles of cellular mechanics in modulating the cell fate and cell differentiation of osteoblasts, but also paves the way for using PCL 3D MEW fibrous substrate cell cultures as an ideal platform to investigate 3D mechanobiology and epigenetics in osteoblasts.

## 2. Materials and Methods

### 2.1. 3D PCL Porous Membranes Fabrication and Characterization

The PCL 3D fibrous substrates with two geometries (random and aligned fibers) were manufactured using medical-grade polycaprolactone (PCL, Purasorb PC 12), as previously described [28]. Briefly, melt electrowriting mode was utilized using a melt electrospinner: spinneret size of 23 G, temperature of 75 °C, an extrusion pressure of 180 kPa, a distance spinneret-collector of 10 mm, a voltage of 8 kV, and a stage speed of 1000 mm/min. The random fiber (PCL-R) fibrous substrates were fabricated by reducing the stage speed to 200 mm/min to allow buckling of the jet to obtain a random pattern. Aligned fibers (PCL-A) were fabricated by collector modifications where the theoretical pore size was 200 µm. The thickness of both PCL-A and PCL-R was approximately 80 µm.

Scanning electron microscopy (SEM; JEOL 7001f, NeoScope JCM-5000 Benchtop SEM, JEOL Pty. Ltd., Sydney, Australia) operating at a voltage of 5 kV was used to characterize the fabricated 3D fibrous substrate morphology following gold coating for 75 s (~10 nm coating thickness). The fiber diameter and pore size were calculated using ImageJ software (1.51V, National Institutes of Health, USA) as described previously [28]. Briefly, SEM images and optical images were taken and fiber center-to-center distance was measured as the pore size, along with fiber diameter.

For mechanical testing, the sample (6 mm long and 10 mm wide) was placed into the grips and tested using a Univert universal tester (Cellscale, Waterloo, Canada) fitted with a 10 N load cell and at a cross-head speed of 5 mm/min at room temperature. Three replicate samples were tested for each design. The tensile modulus was calculated from the initial linear section of the tensile curve from stain ranging from 1–8%).

### 2.2. Human Primary Osteoblast Culture and Differentiation

The isolation and culture of primary human alveolar bone-derived osteoblasts (hOBs) were performed according to published protocols [29,30,31]. All participants involved who underwent third molar extraction gave informed consent and the Human Ethics Committees of the University of Queensland approved the research protocol (Approval number 2019000134). hOBs were cultured in Dulbecco’s modified Eagle’s medium (DMEM; Gibco-Invitrogen) supplemented with 10% vol/vol fetal bovine serum (FBS; Thermo Scientific Australia, Sydney, Australia) and 50 U/mL penicillin and 50 mg/mL streptomycin (P/S; Gibco-Invitrogen) at 37 °C in a humidified CO_2_ incubator. For osteogenic differentiation, cells were cultured in an osteogenic DMEM medium containing 10% FBS, 50 µg/mL ascorbic acid, 3 mM β-glycerophosphate, and 10 nM dexamethasone (Sigma-Aldrich, St. Louis, MO, USA). Cells sourced from two patients at passages 4–5 were used for all experiments. Cells cultured on polystyrene tissue culture plates (TCP) were used as 2D controls.

3D PCL fibrous substrates were treated with 2 N sodium hydroxide (NaOH) for 1 h before coating with FBS in order to enhance cell seeding efficacy, as described previously [32].

For immunostaining and global DNA epigenetics, the cells were seeded at a density of 3000 cells per 1 × 1 cm^2^ porous membrane for 24 h.

### 2.3. Immunofluorescence Staining

Immunofluorescent staining was performed as previously described [8,33,34]. Briefly, hOBs were fixed in 4% PFA at room temperature for 10 min and then permeabilized with 0.05% Triton X-100 in PBS containing 320 mM sucrose and 6 mM magnesium chloride. After blocking with 1% bovine serum albumin, 0.1% Tween-20, 0.3 M glycine, 10% goat serum (Gibco) in PBS for 1 h, primary antibodies (Vinculin, 1:400, sc-73614, Santa Cruz Biotechnology; Lamin A/C, 1:200, sc-376248, Santa Cruz Biotechnology; YAP, sc-101199, Santa Cruz Biotechnology, Dallas, TX, USA) were incubated for 1 h at room temperature. After three PBS-Tween 20 washes, AlexaFluor-488 [H+L] secondary antibodies supplemented with AlexaFluor-conjugated phalloidin and DAPI were added for 1 h at room temperature. Images (>15 cells) were acquired with a Nikon confocal microscope (Nikon, Tokyo, Japan).

### 2.4. Focal Adhesion, Nucleus and Nuclear YAP Data Analysis

For morphological analysis of focal adhesion, vinculin staining images (>15 cells) were processed and analyzed in Fiji-ImageJ software (1.51V, National Institutes of Health, USA, v1.8.0). In this analysis, FAs were detected as bright clusters of vinculin, with FA numbers, percentage of FAs containing F-actin and FAs length [8]. Nucleus area, circularity, and aspect ratio (major axis/minor axis) were calculated from LAMIN A/C images using ImageJ software. Nuclear YAP/TAZ (%) were scored as predominantly nuclear versus evenly distributed/predominantly cytoplasmic, as previously described [8].

### 2.5. DNA Isolation and Global DNA Methylation Analysis

Genomic DNA was extracted using the PureLink™ Genomic DNA Mini Kit (Invitrogen™, ThermoFisher Scientific, Sydney, Australia) according to the manufacturer’s instructions. Briefly, the cells from the various groups (three replicates) were suspended in lysis buffer and RNase A and Proteinase K were added to remove the protein and RNA. The quality and quantity of the DNA were determined by a NanoDrop One spectrophotometer (ThermoFisher Scientific, Waltham, MA, USA).

Global methylation analysis of 5 methyl cytosine (5mC), 5-hydroxymethylcytosine (5hmC), and N 6-methyladenosine (m6A) for DNA was performed by using a Global DNA Methylation Assay Kit (5mC, ab233486, Abcam, Cambridge, UK) Global DNA Hydroxymethylation Assay Kit (5hmc, ab233487, Abcam, Cambridge, UK), and an m6A DNA Methylation Assay Kit (ab233488, Abcam, Cambridge, UK), as per the manufacturer’s instructions. Briefly, sample DNA positive controls at six different concentrations (to generate a standard curve) and negative control were mixed with DNA binding solution and incubated at 37 °C for 60 min. After washing three times with 150 µL washing buffer, 5mC/5hmC/m6 antibodies, along with a signal indicator and enhancer solution, were added and incubated at room temperature for 1 h. After washing with wash buffer five times, 50 µL developer solution was added and incubated for 3 min at room temperature until the positive control with the highest concentration turned blue. Subsequently, 50 µL of stop solution was added to each well for 2 min to stop the enzyme reaction. The absorbance was measured at 450 nm within 2 min on an Infinite Pro spectrometer.

The global methylation level of all DNAs was calculated using the following equations as described previously [20]:

(1) $$5\mathrm{mc}/5\mathrm{hmC}\;\%=\frac{\mathrm{Sample}\;\mathrm{OD}\;-\;\mathrm{Negative}\;\mathrm{Control}\;\mathrm{OD}}{\mathrm{Slope}\;\times \;S\;}\times 100\%$$

(2) $$m6A\;\%=\frac{\left(\mathrm{Sample}\;\mathrm{OD}\;-\;\mathrm{Negative}\;\mathrm{Control}\;\mathrm{OD}\right)/S\;}{\left(\mathrm{Positive}\;\mathrm{Control}\;\mathrm{OD}\;-\;\mathrm{NDC}\;\mathrm{OD}\right)/P}\times 100\%$$

where, the slope (OD/1%) was determined from the standard curve using linear regression; S is the amount of input sample DNA in ng; P is the amount of positive input control in ng; OD is the optical density.

### 2.6. RNA Isolation and Quantitative Real-Time Polymerase Chain Reaction (qPCR)

Quantitative reverse transcription-polymerase chain reaction (RT-qPCR) was used to measure the mRNA expression of osteogenic markers: *alkaline phosphatase (ALP)*, *osteopontin (OPN)*, *runt-related transcription factor 2 (RUNX2), OSX,* and *BSP*. The primers are listed in Table 1. Total RNA was isolated using a PureLink™ RNA Mini Kit with on-column DNase treatment (Invitrogen™, ThermoFisher Scientific, Sydney, Australia) according to the manufacturer’s instructions. cDNA was synthesized from 200 ng RNA using a First Strand cDNA Synthesis Kit (ThermoFisher Scientific, Sydney, Australia). Quantitative PCR (qPCR) reactions were prepared in a total volume of 10 µL with PowerUp SYBR Green Master Mix (ThermoFisher Scientific, Sydney, Australia) and 0.1 mM forward and reverse primers. StepOnePlus PCR equipment (Applied Biosystems) was used to run the samples, with 2 min at 95 °C, then 40 cycles of 3 s at 95 °C and 30 s at 60 °C, followed by a melt curve. Relative mRNA expression was analyzed using the 2^−ΔCT^ method, after being normalized with two housekeeping genes (18s rRNA and GAPDH).

### 2.7. Alizarin Red S Staining

For the differentiation assay, cells were seeded at a density of 10,000 cells per 1 × 1 cm^2^ fibrous substrates for three weeks before Alizarin Red S staining for calcium mineral formation from three replicates. Samples were incubated with a 40 mM Alizarin Red S solution (pH 4.1) for 15 min and images were taken using a Nikon microscope. Then, 10% of cetylpyridinium chloride (200 µL) was used to dissolve the staining and the absorbance was measured at 540 nm in an Infinite Pro spectrophotometer. 

The DNA content of each sample was measured using a PicoGreen dsDNA quantitation kit (Invitrogen), according to our previous work [28]. Alizarin Red S absorbance was normalized with DNA content from each well to further measure the relative calcium content.

### 2.8. Statistical Analysis

All data are displayed as the mean ± standard deviation (SD). The statistical differences between TCP, PCL-R, and PCL-A were determined in GraphPad Prism 9.0 software (GraphPad Software, San Diego, CA, USA) using a one-way analysis of variance (ANOVA) followed by Tukey’s multiple comparison tests. A *p* < 0.05 was considered statically significant.

## 3. Result and Discussion

### 3.1. Focal Adhesions Are Altered on Aligned Fibers on a PCL Porous Scaffold

The majority of the current literature has investigated the effect of 2D biomaterial cues (such as topography) on understanding cell behaviour via mechanosensing and epigenetics on a given cell type [35,36,37]. There is limited knowledge of how fiber alignment regulates cellular/nuclear mechanosensing and epigenetics in osteoblasts. The present study successfully fabricated a PCL fibrous scaffold with aligned and random 3D topographical microenvironments for osteoblast cellular and nuclear mechanics by the additive manufacturing, MEW, fabrication technique.

**Figure 1:**
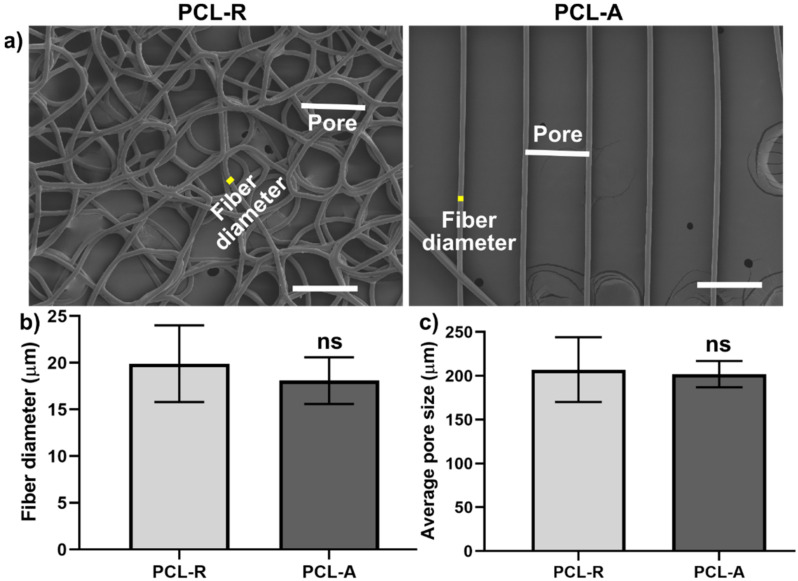
SEM images (**a**), fiber and pore size quantification (**b**,**c**) of electrospun 3D polycaprolactone (PCL) porous scaffold with different geometries using the melt electrospinning direct writing mode. Scale bar in a: 200 µm. ns: no significant difference between PCL-R and PCL-A groups.

SEM images (Figure 1a) demonstrate the 3D PCL fibrous scaffolds with random (PCL-R) and aligned (PCL-A) fibers fabricated by MEW. As the fabrication parameters were consistent, the fiber diameters of random and aligned fibers did not show any significant changes (*p* < 0.05). The PCL-R diameter size was 19.9 ± 4.1 µm, whereas PCL-A was 18.1 ± 2.5 µm (Figure 1b). The pore size (distance between fibers from center to center) was comparable between both random and aligned fibers (PCL-R: 207 ± 39 µm; PCL-A: 202 ± 15 µm). The obtained fiber diameters results were in good agreement with our previous work [28]; however, the PCL-A group’s pore size was smaller than ours [28]. This may be caused by technical variations during different manufacturing batches, while our PCL-R and PCL-A had similar pore sizes (~200 µm) since scaffold pore size is one of the critical factors directing adhesion and differentiation [38,39]. Additionally, mechanical testing demonstrated that the randomly organized membranes were softer than the aligned fibers with a respective tensile modulus of 32.5 ± 0.4 kPa and 56.7 ± 22.5 kPa, respectively. Aside from the osteoblasts investigated in the current study, these 3D platforms can be applied to other cell types for mechanotransduction and epigenetics research.

**Figure 2:**
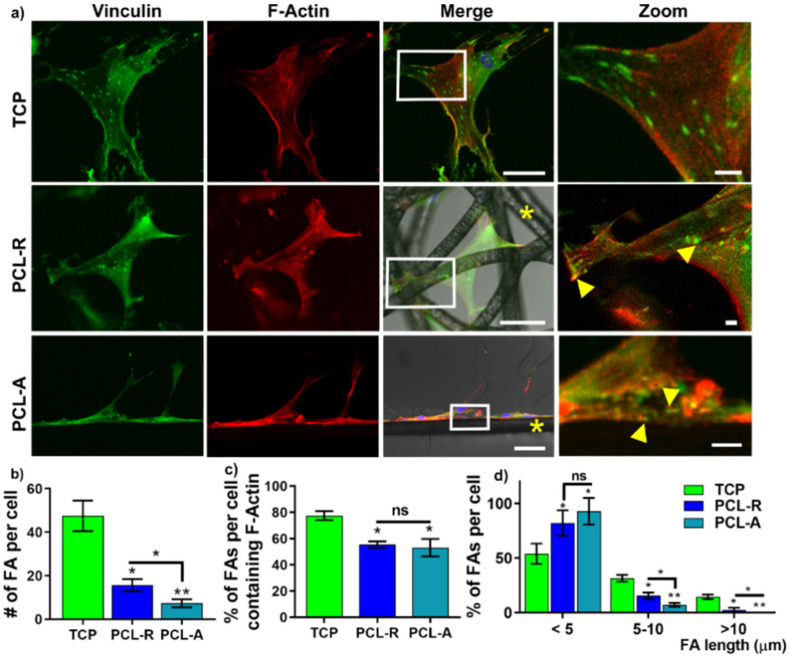
Osteoblast cell shape and focal adhesion on the 3D PCL groups and 2D TCP at 24 h. Representative confocal images (**a**) and quantification (**b**–**d**) of vinculin in hOBs (>15 cells per group per donor). * PCL fibers; arrows: focal adhesions (FAs); Scale: 50 µm in low-magnification, 10 µm in zoom. * *p* < 0.05, ** *p* < 0.02.

Given that cell morphology drastically changes in a 3D microenvironment vs. 2D substrate, the question arises as to whether the molecular mechanisms of 3D mechanotransduction also differ. The architecture of adhesion complexes in 3D matrices is remarkably different from those of cells in 2D TCP cultures [40], which is in line with the results in the current study. Although nano-scaled FAs (i.e., a key FA protein and ‘molecular clutch’–vinculin [41]) composition is comparable between 2D and 3D microenvironment, actin fibers and FAs in 3D are reduced and smaller compared to the 2D situation [42]. The primary hOBs were cultured on 2D (tissue culture plate-TCP) and 3D geometries (random and aligned PCL porous membranes coated with fetal bovine serum) for 24 h. The data demonstrated a significant cell shape difference between the groups (spread on PCL-R and TCP groups, elongated on PCL-A group). The FAs in hOBs on 3D (both random and aligned) porous scaffolds were mainly detected at the corners or sharp angles formed by adjacent melt electrospun written fibers (yellow arrows), with significantly reduced vinculin (a key focal adhesion protein) numbers (Figure 2b), fewer FAs containing F-actin fibers (Figure 2c), and mature vinculin (>10 µm; Figure 2d) when compared to the 2D TCP substrate. Furthermore, there were more FAs numbers and less mature FAs in hOBs on the random porous membranes than the aligned porous membranes (Figure 2b,d).

Our data demonstrated that the 3D microenvironment (for both random and aligned PCL porous scaffold) led to significantly reduced numbers and smaller vinculin FAs in human osteoblasts compared to 2D TCP (Figure 2), which was in agreement with previous reports although using 3D hydrogels [40,42,43]. Whereas FAs adopted a spindle shape on the 2D TCP, our data showed that 3D FAs were round-shaped (Figure 2a) and were mainly located in the vicinity of optus angles between the PLC fibers, which is consistent with data from 2D micropatterning [44]. Fewer FAs numbers (Figure 2b) and elongated nucleus (Figure 2a) were detected in the aligned PCL porous membranes, given that the cell and nucleus need to accommodate the change in cell shape, which is in accordance with cell shape in aligned nano-PCL fibers [22,44,45]. It is worth noting that cells may interact with multiple fibers at the corner of the PCL-A scaffold, while most of the cells might adhere to a single fiber in the PCL-A group with less surface area than random fibers where cells can interact with more than one fiber.

### 3.2. The Effect of Fiber Alignment on Nucleus Mechanosensing

**Figure 3:**
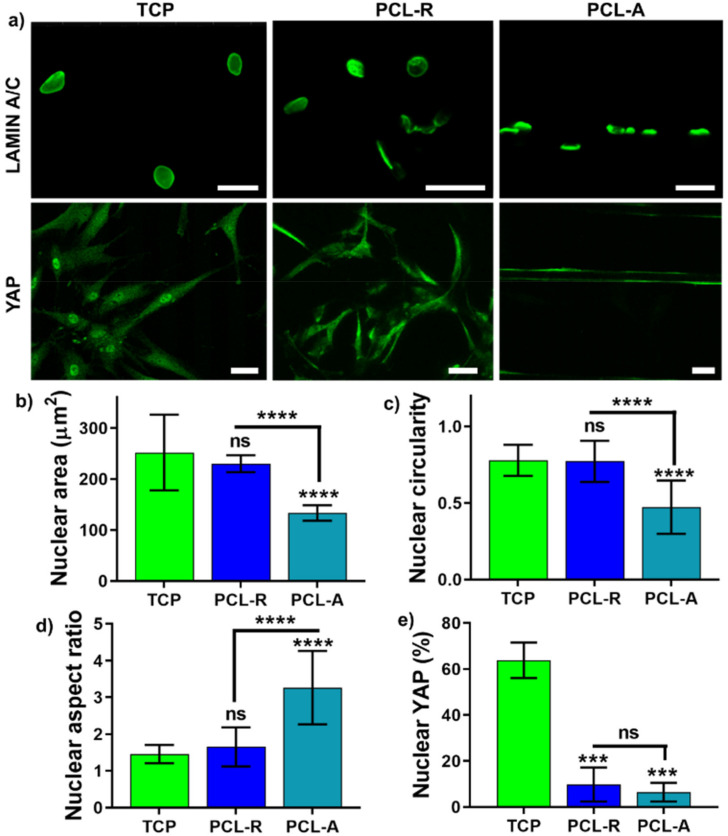
Nuclear LAMIN A/C, yes-associated protein (YAP) expression (**a**) and quantification of nuclear area (**b**), circularity (**c**), aspect ratio (**d**) and nuclear YAP localization (%) (**e**) in hOBs after 24 h. Scale in (**a**): 50 µm. *** *p* < 0.001, ****, *p* < 0.0001.

Besides the cellular mechanosensing, the nucleus is mechanosensitive and linked mechanically to the extracellular matrix via the cytoskeleton that transmits forces to the nuclear envelope (via LAMIN A/C protein) and mediates the cell’s genome and epigenome [12,46]. Our data demonstrate that LAMIN A/C was detected in both 2D and 3D, although the aligned fibers led to an elongated nucleus with enhanced LAMIN A/C fluorescence (Figure 3a). YAP is a known key mechanotransductive transcription factor that contributes to the retention storage of the mechanical ‘memory’ of past cell–matrix interactions [11]; our previous research showed that in 2D substrates, higher vinculin expression led to higher nuclear YAP accumulation and nuclear mechanical tension at an early point (24 h) and was associated with increased osteogenic differentiation in human mesenchymal stem cells [8,45]. LAMIN A/C and YAP were stained for hOBs on the 3D porous scaffolds and TCP (Figure 3). Higher nuclear LAMIN A/C expression with a significantly smaller, elongated nucleus was found on the aligned PCL fibrous substrates, where nuclear shape, area, circularity, and aspect ratio were similar between the PCL-R and TCP groups. It was noted that YAP was mainly localized in the nucleus in 2D culture, while there was a significantly reduced nuclear YAP (%) expression in both 3D groups (Figure 3a,e), suggesting that the nucleus is “softer” in the 3D PCL porous membranes compared to the 2D TCP.

It has previously been demonstrated that nuclear YAP/TAZ interacts with the TEAD transcription factor and recruits the nucleosome remodelling deacetylase complex on target genes, causing histone deacetylation [47]. However, it is unclear whether YAP localization would alter DNA epigenetics, in terms of global 5mC, 5hmC, and m6dA methylation.

### 3.3. DNA Epigenetics Is Modulated by Fiber Alignment

**Figure 4:**
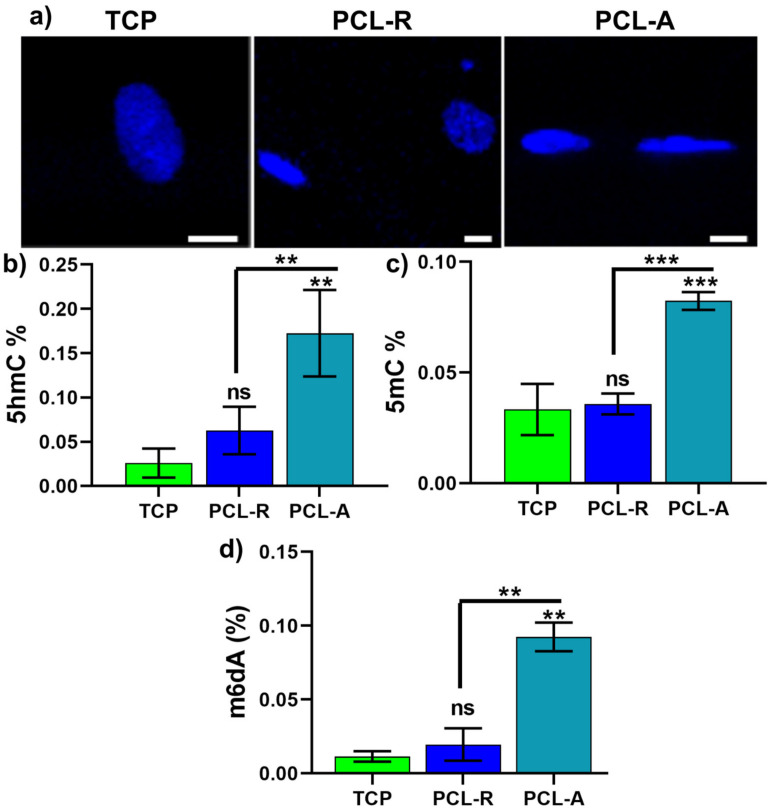
Representative nuclei (**a**), global 5mC (**b**), 5hmC (**c**), and m6dA (**d**) methylation changes in hOBs on different fiber aligned membrane after 24 h. Scale in (**a**): 50 µm. m6dA: N-6 methylated deoxyadenosine; 5mC: 5-Methylcytosine; 5hmC: 5-Hydroxymethylcytosine. Data: mean ± SD. ** *p* < 0.02, *** *p* < 0.001.

To explore the link between nuclear stiffness and global methylation, the effect of different fiber alignment on 5mC, 5hmC, and m6dA expression in hOBs was determined at 24 h. The comparison between random and aligned fibers showed that a mixture of spread and round cell nucleus was observed in the PCL-R group, while the PCL-A group led to a stretched cell with an elongated nucleus (Figure 4a), which is in line with cells cultured on random and aligned chemically cross-linked gelatin fibers in a PCL fibrous substrate [45].

Global methylation results indicate no significant difference between TCP and the random group in global 5mC, 5hmC, and m6dA methylation. However, the aligned PCL fibers led to increased global hypermethylation for 5mC (Figure 4b), 5hmC (Figure 4c), and m6dA (Figure 4d) compared to both 2D TCP and PCL-R groups.

### 3.4. Cell Differentiation on Different Fiber Aligned Porous Membranes

**Figure 5:**
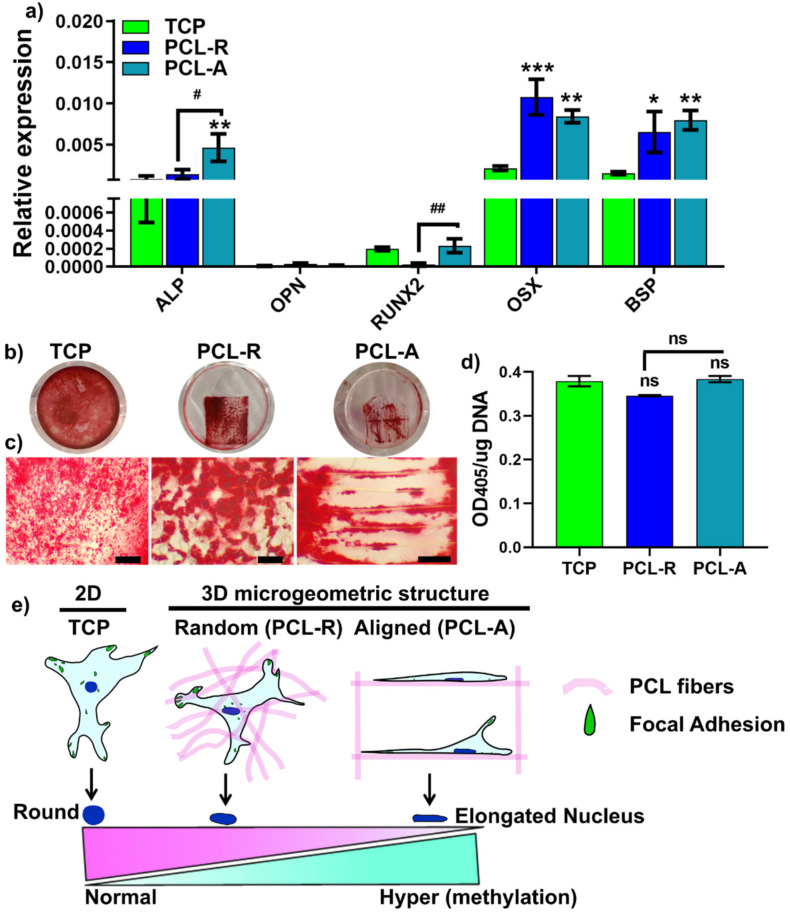
Gene expression of osteogenic markers (**a**) after 1-week osteogenic culture. * *p* < 0.05, ** *p* < 0.002; *** *p* < 0.0002 vs. TCP, # *p* < 0.05, ## *p* < 0.002 between PCL-R and PCL-A. Alizarin Red staining (**b**,**c**) and quantification (**d**) of hOBs after 3-week osteogenic differentiation in random or aligned PCL porous scaffolds. Scale for image (**b**): 50 µm. ns: no significant difference. (**e**) Schematic illustration of osteoblast response to fiber alignment via altered focal adhesion, nuclear mechanosensing, and global methylation.

Our previous research showed that in 2D substrates, higher vinculin expression led to higher nuclear YAP accumulation and nuclear mechanical tension at an early point (24 h), with increased long-term (3-week) osteogenic differentiation in human mesenchymal stem cells [8,45]. Our data showed that 3D PCL scaffolds led to significantly decreased FAs (Figure 1a) and nuclear YAP localization (Figure 3a,e), compared to the 2D groups. Long-term osteogenic differentiation was performed on PCL-R, PCL-A, and TCP groups for one and three weeks in osteoinductive media. Osteogenic markers (*ALP*, *OPN*, *RUNX2*, *OSX*, *BSP*) on different substrates were performed by RT-qPCR after 1-week osteogenic differentiation. The results showed that PCL-A group had significantly increased gene expression of osteogenic markers such as *ALP*, *OSX*, and *BSP* compared to 2D TCP. Moreover, *ALP* and *RUNX2* were significantly enhanced in the PCL-A group in comparison to the PCL-R group. However, in contrast to the gene expression patterns, Alizarin Red S staining showed that all three groups enabled the osteogenic differentiation in hOBs (Figure 5b,c) and the quantification of the ARS normalized by DNA content did not result in any significant difference (Figure 5d).

In 2D substrates, it is well-known that higher nuclear YAP and FAs are associated with enhanced osteogenic differentiation and mineralization in MSCs [8], but it is unclear whether this applies to 3D microenvironments in osteoblasts. Our results showed a different trend that lower nuclear YAP and fewer FAs (24 h post-seeding) in the PCL-A group led to higher gene expression of early osteogenic markers (7-day post osteogenic differentiation), with no change in calcium mineralization after 3-week osteogenic induction. This contrary trend between mechanotransduction and osteogenic differentiation may explain that osteogenic differentiation and mineralization may be modulated by other complex mechanisms, which requires more studies to explore the underlying mechanisms.

The study has its limitations: (1) different fiber (nano- and micro-scaled) and pore size (20–200 µm) of 3D mPCL scaffolds were not included to compare cell behaviour; and (2) more assays are required to confirm the osteogenic capability of 3D PCL scaffolds. Future studies with various fiber diameters, pore sizes, and more differentiation assays should be considered.

Taken together, the results indicate that aligned fibers within 3D PCL fibrous substrates lead to reduced nano-scaled focal adhesion and nuclear YAP localization compared to 2D TCP. Furthermore, aligned 3D fibers lead to an elongated cell and nuclear shape, resulting in hypermethylation of 5mC, 5hmC, and m6dA compared to random fibers. This altered cellular/nuclear mechanosensing and DNA epigenetics may provide new insights into the endpoint differentiation outcome between 3D random and aligned porous membranes (Figure 5e). However, more studies using cutting-edge techniques such as methylated DNA immunoprecipitation (MeDIP) next-generation sequencing are required for investigating the whole methylome signature. Hence, changing the local microenvironment via different fiber alignment may be an effective strategy for altering nuclear stiffness and epigenetics in hOBs.

## 4. Conclusions

Although there is still much unknown about how biophysical cues of random and aligned 3D fibrous substrates can impact nuclear signaling, the present study may advance our understanding of random and aligned fibers modulating the epigenetic regulation of osteoblast cells through mechanotransduction. In addition, our findings suggest that fiber alignment acts as an epigenetic factor that affects cell and nuclear mechanosensing, and extended exposure to defined geometric microenvironments may lead to changes in the hOBs phenotype and fate determination of osteoblast cells. Our results highlight the importance of understanding how 3D geometric physical aspects with random and aligned fiber physical cues can alter cellular/nuclear nano-mechanics and DNA epigenetics. Future studies using other cell types are required to determine how 3D mechanotransduction may be harnessed to develop novel biomaterials for tissue engineering and regenerative medicine.

## Figures and Tables

**Table 1:** Primers used in this study.

	Forward Primer (5′-3′)	Reverse Primer (5′-3′)
***ALP***	TCAGAAGCTAACACCAACG	TTGTACGTCTTGGAGAGGGC
***OPN***	TCACCTGTGCCATACCAGTTAA	TGAGATGGGTCAGGGTTTAGC
***RUNX2***	GGAGTGGACGAGGCAAGAGTTT	AGCTTCTGTCTGTGCCTTCTGG
***OSX***	GCAAAGCAGGCACAAAGAAG	CAGGTGAAAGGAGCCCATTAG
***BSP***	AGGCTGAGAATACCACACTTTC	GGATTGCAGCTAACCCTGTAT
***18s***	TTCGGAACTGAGGCCATGAT	CGAACCTCCGACTTCGTTC
***GAPDH***	TCAGCAATGCATCCTGCAC	TCTGGGTGGCAGTGATGGC

## Data Availability

The data presented in this study are openly available online.
